# Older Men Who Use Computers Have Lower Risk of Dementia

**DOI:** 10.1371/journal.pone.0044239

**Published:** 2012-08-28

**Authors:** Osvaldo P. Almeida, Bu B. Yeap, Helman Alfonso, Graeme J. Hankey, Leon Flicker, Paul E. Norman

**Affiliations:** 1 School of Psychiatry & Clinical Neurosciences, University of Western Australia, Perth, Western Australia, Australia; 2 Western Australian Centre for Health & Ageing, Centre for Medical Research, University of Western Australia, Perth, Western Australia, Australia; 3 Department of Psychiatry, Royal Perth Hospital, Perth, Western Australia, Australia; 4 School of Medicine and Pharmacology, University of Western Australia, Perth, Western Australia, Australia; 5 Department of Endocrinology and Diabetes, Fremantle Hospital, Fremantle, Western Australia, Australia; 6 Department of Neurology, Royal Perth Hospital, Perth, Western Australia, Australia; 7 Department of Geriatric Medicine, Royal Perth Hospital, Perth, Western Australia, Australia; 8 School of Surgery, University of Western Australia, Perth, Western Australia, Australia; Federal University of Rio de Janeiro, Brazil

## Abstract

**Objective:**

To determine if older men who use computers have lower risk of developing dementia.

**Methods:**

Cohort study of 5506 community-dwelling men aged 69 to 87 years followed for up to 8.5 years. Use of computers measured as daily, weekly, less than weekly and never. Participants also reported their use of email, internet, word processors, games or other computer activities. The primary outcome was the incidence of ICD-10 diagnosis of dementia as recorded by the Western Australian Data Linkage System.

**Results:**

1857/5506 (33.7%) men reported using computers and 347 (6.3%) received a diagnosis of dementia during an average follow up of 6.0 years (range: 6 months to 8.5 years). The hazard ratio (HR) of dementia was lower among computer users than non-users (HR = 0.62, 95%CI = 0.47–0.81, after adjustment for age, educational attainment, size of social network, and presence of depression or of significant clinical morbidity). The HR of dementia appeared to decrease with increasing frequency of computer use: 0.68 (95%CI = 0.41–1.13), 0.61 (95%CI = 0.39–0.94) and 0.59 (95%CI = 0.40–0.87) for less than weekly, at least weekly and daily. The HR of dementia was 0.66 (95%CI = 0.50–0.86) after the analysis was further adjusted for baseline cognitive function, as measured by the Mini-Mental State Examination.

**Conclusion:**

Older men who use computers have lower risk of receiving a diagnosis of dementia up to 8.5 years later. Randomised trials are required to determine if the observed associations are causal.

## Introduction

As the World's population ages, the number of people experiencing cognitive decline and dementia will continue to increase. Currently available estimates suggest that over 24 million people worldwide had dementia in 2005, with this number expected to reach 50 million by 2025 [1]. The direct and indirect costs associated with dementia will also continue to rise, and conditions such as Alzheimer's disease are expected to become leading causes of health expenditure in developed and developing countries [2]. Such considerations have stimulated the search for factors that might delay or prevent the progression of cognitive decline in older adults at risk, with promising results being reported for physical activity [3], adequate management of diabetes and hypertension [4], [5], and participation in cognitively stimulating activities [6]. Data from the Bronx Aging Study showed that the hazard of dementia over 5 years was decreased amongst older adults involved in cognitively stimulating activities, with the lowest risk observed for the most active participants [6]. A subsequent randomized trial of cognitive training for adults aged 65–94 years (ACTIVE trial) found that the 10-week intervention was associated with specific cognitive gains over 2 years [7], whereas reasoning training led to less pronounced decline in self-reported instrumental activities of daily living over 5 years [8]. Although the ACTIVE trial was unable to establish if the intervention decreased the onset of dementia amongst participants, its results are consistent with the hypothesis that regular involvement in mentally demanding activities improves function and may reduce the risk of dementia.

In this context, the increasing ease of access to personal computers that has occurred over the past 20 years offers hope that the growing exposure of older adults to this technology will enhance their participation in mentally stimulating activities and contribute to maintain cognitive function and reduce the prevalence of dementia in the community. In Australia, 47% of the population over the age of 60 years used computers in 2009, compared with only 29% in 2003 [9]. In Western Australia, 66% of older adults reported having a computer at home in 2009, and 58% had access to the internet [9]. Over 80% of those with access to the internet used email and chat sites or conducted general browsing. About 50% used the internet to pay bills, manage their finances or to access government services. Over 1/3 purchased goods and about 10% used the internet to manage their shares [9]. However, the health effects of computer use and internet access remains uncertain.

As the use of computers has previously been associated with improved cognitive function in adulthood and old age [10] and participation in cognitively stimulating activities reduces the long-term risk of dementia [6], [11], we hypothesized that older computer users would have lower risk of developing dementia than non-users over a follow up period of up to 8 years. We conducted this study to test this hypothesis.

## Methods

### Ethics statement

The study was conducted according to the principles expressed in the Declaration of Helsinki, and the Human Research Ethics Committee of the University of Western Australia approved the study protocol and all men provided written informed consent to participate.

### Study design and participants

This study used a longitudinal population-based sample of older men living in the Perth metropolitan area, the Health In Men Study (HIMS). Details regarding the recruitment of participants have been described elsewhere [12]. Briefly, we recruited a community-representative sample of 19352 Australian men aged 65 to 85 years living in the Perth metropolitan area between 1996 and 1998, of whom 12203 completed the first assessment of HIMS. Five years later 5583 of the 10940 surviving men agreed to participate in a follow up assessment that included questions about the use of computers (HIMS wave 2, 2001 to 2004). Of these, 30 men were excluded from further participation in this study because they had a recorded diagnosis of dementia in the Western Australian Data Linkage System (WADLS) prior to the date of their assessment (prevalent cases – please see details about the diagnosis of dementia below). Another 47 men did not answer the questions regarding the use of computers and were also excluded, leaving a study sample of 5506 participants.

### Outcome of interest: dementia

The primary outcome of interest of the study was a recorded diagnosis of a dementia syndrome in WADLS for the first time after the HIMS wave 2 assessment. WADLS brings together name-identified records for all in-patient hospital admissions as well as public sector mental health services (in-patient, out-patient and community mental health services), and includes all morbidity and mortality data of Western Australia coded according to the International Classification of diseases tenth revision (ICD-10) and, for events that occurred before 1996, the ICD-9. The validity of these data linkage is well established [13], [14]. The diagnosis of dementia was the primary endpoint of interest of the study, and was defined according to the following ICD-9 and ICD-10 codes: all listed diagnoses (primary and secondary) for Alzheimer's dementia 331.0, F00, G30; Vascular dementia 290.4, F01; fronto-temporal dementia 331.1, F02.0, G31.0; Huntington's disease 333.4, G10, F02.2; Parkinson's dementia or dementia with Lewy bodies F02.3, 331.82; and non-specific dementia 290.0, 290.1, 290.2, 290.3, 290.8, 290.9, 294.1, 294.8, 331.2, F02.8, F03, F05.1, G31.1, G31.8, G31.9. To improve case ascertainment, the text terms of the above conditions were also searched in the morbidity and mortality data systems, along with the following alternative medical terms: multi-infarct dementia, arteriosclerotic dementia, fronto-temporal lobe dementia, primary progressive dementia, corticobasal dementia, and Pick's dementia. As the accuracy of specific diagnostic causes of dementia in WADLS is uncertain, we opted to group all entries under the general heading of ‘dementia’. In addition, we retrieved from WADLS information about the dates of all contacts associated with a diagnosis of dementia and considered the date of onset to be the same as the date of the first recorded event.

### Explanatory variables

During HIMS wave 2, we asked participants: ‘How often do you use a personal computer?’ Possible answers were ‘never’, ‘every day’, ‘at least every week’, ‘less than every week’. We classified participants who answered ‘never’ as ‘computer no-users’ and those who offered any of the other three answers as ‘computer users’. Computer users were also asked: ‘What do you use a personal computer for?’ Participants could choose one or more of the following answers: word processing, internet, email, games, other.

We calculated the age of participants as the difference in days between the date of the assessment for HIMS wave 2 and their date of birth divided by 365.25. In addition, men reported the highest level of education achieved (completed or not high school) and their country of birth. Participants completed the rating of the Duke Social Support Index, which is a valid measure of network support [15]. For the purposes of this study, we assessed three aspects of network support: number of times in the past week spent with somebody who does not live in the same house, number of times in the past week that participant talked to somebody on the phone, and number of times in the past week that participant went out for meetings or social gatherings. Possible answers ranged from zero to 7 (i.e., every day), yielding a maximum total score of 21. We also used the Index of Relative Socio-Economic Disadvantage (IRSED), which is a component of the Socio-Economic Indexes for Areas (SEIFA) of Australia [16]. Lower IRSED rankings indicate greater socio-economic disadvantage [16].

Participants also reported whether they had trouble seeing newspaper print even with glasses (yes/no) and hearing a conversation even with a hearing aid (yes/no). Those who answered ‘yes’ to any of these two questions were considered to show evidence of sensory impairment. They completed the 15-item Geriatric Depression Scale (GDS-15) during HIMS wave 2 and, *a priori*, those with a total score of 7 or more were considered to display clinically significant depressive symptoms at the time of assessment. This relatively high cut-point was chosen to ensure high specificity for the diagnosis of depression in this sample [17]. We used administrative medical information from WADLS during the 10 years prior to assessment at HIMS wave 2 to calculate the Charlson index and determine the presence of significant medical comorbidity in our sample [18]. The index takes into account 17 common medical conditions that predict 1-year mortality: myocardial infarction, congestive heart failure, peripheral arterial disease, cerebrovascular disease, dementia, chronic pulmonary disease, connective tissue disease, ulcer disease, liver disease, diabetes (including diabetes with end organ damage), hemiplegia, renal disease, leukemia, lymphoma, other tumours, metastatic tumours and AIDS. Charlson and colleagues used adjusted relative risks to assign integer weights to these conditions within a composite index score that ranges from 0 to 37. Coding algorithms to define comorbidities followed the procedures described by Quan *et al*. [19] and scores were calculated using Stagg's Charlson's index Stata 9.2 routine (StataCorp, College Station, Texas). We stratified scores to reflect the increasing severity of comorbidity associated with the index, and considered that men with a score of 3 or more showed evidence of significant medical morbidity [20].

During the second wave of the study, a subsample of 3888 men agreed to complete the Mini-Mental State Examination (MMSE), which is a brief neuropsychological battery that assesses cognitive function [21]. Total scores range from 0 to 30, with higher scores indicating better cognitive function [22].

### Statistical analyses

Data were analyzed with the statistical package Stata release 12.1 (StataCorp, College Station, TX). We used descriptive statistics (mean, standard deviation of the mean [SD], proportions) to summarise our data, and the crude odds ratio (OR) as a measure of the cross-sectional association between computer use and demographic, social and clinical variables. We estimated the annual incident ratio (IR) of dementia per 1000-person years via the natural exponentiation of the coefficients derived from Poisson regression and used Cox regression to calculate the crude and adjusted hazard ratio (HR) of dementia in the HIMS cohort according to the use of computers, censoring the data at the time participants had a health contact where they received the diagnosis of dementia or when they died, whatever came first. The last follow up date available for participants was the 29^th^ September 2009. The analyses conformed to the assumption of proportionality. We employed the same approach to investigate the HR of dementia according to the various computer uses, and logistic regression to investigate the crude and adjusted association between the use of computers and cognitive impairment (OR)(time to event in this case was not known). Confounders included in the analyses were age (in years), education, social network (lowest vs medium + highest tertiles), depression, and weighted Charlson index (<3 vs ≥3).

We used multiple imputation strategies to estimate the MMSE score of men who did not complete the cognitive evaluation during HIMS wave 2, and multiple imputation estimates to calculate the adjusted HR of dementia taking into account the MMSE score of participants at the time of their assessment. We created 10 files to calculate our risk estimates.

All risk estimates are reported alongside their 95% confidence interval (95%CI). Alpha was set at 5% and all statistical tests were two-tailed.

## Results

The mean age of our 5506 men was 75.5 years (SD = 4.2) at the time of their HIMS wave 2 assessment. Of those, 1857 (33.7%) reported using computers. Computer users were younger than non-users (74.7±3.9 vs 75.9±4.2, t = 10.52, p<0.001) and had higher MMSE scores (27.0±2.2 vs 26.1±2.6; t = 11.69, p<0.001; n = 2383 and 1505 respectively). Table 1 summarises the characteristics of participants at that point in time. The proportion of participants using computers decreased with increasing age, and was more frequent amongst men who had completed at least high school. Computer users had a more active social network and were less likely than non-users to show evidence of clinically significant depressive symptoms (i.e., GDS-15≥7) or medical morbidity (weighted Charlson index ≥3).

**Table 1:** Basic demographic, social and clinical characteristics of older men according to their use of computers.

	Computer non-user N = 3,649 n (%)	Computer user N = 1,857 n (%)	Odds Ratio (OR) (95%CI)
Age group (years) 70–74	1569 (42.5)	1041 (56.1)	1
75–79	1303 (35.3)	560 (30.2)	0.66 (0.58–0.75)
80–84	654 (17.9)	219 (11.8)	0.50 (0.42–0.60)
85+	141 (3.9)	36 (1.9)	0.38 (0.26–0.56)
Completed high school education	1371 (37.6)	1170 (63.0)	2.83 (2.52–3.18)
Not born in Australia	1422 (39.0)	752 (40.5)	1.07 (0.95–1.20)
Social network: medium or top tertile	2327 (64.0)	1389 (75.0)	1.69 (1.49–1.92)
IRSED: medium or top tertile	2369 (66.3)	1246 (68.4)	1.10 (0.98–1.24)
Sensory impairment	1353 (37.1)	683 (36.8)	0.99 (0.88–1.11)
Clinically significant depression	260 (7.3)	64 (3.5)	0.46 (0.35–0.61)
Charlson index: 3 or more morbidities	569 (15.6)	239 (12.9)	0.80 (0.68–0.94)

IRSED: Index of Relative Socio-Economic Disadvantage.

**Figure 1:**
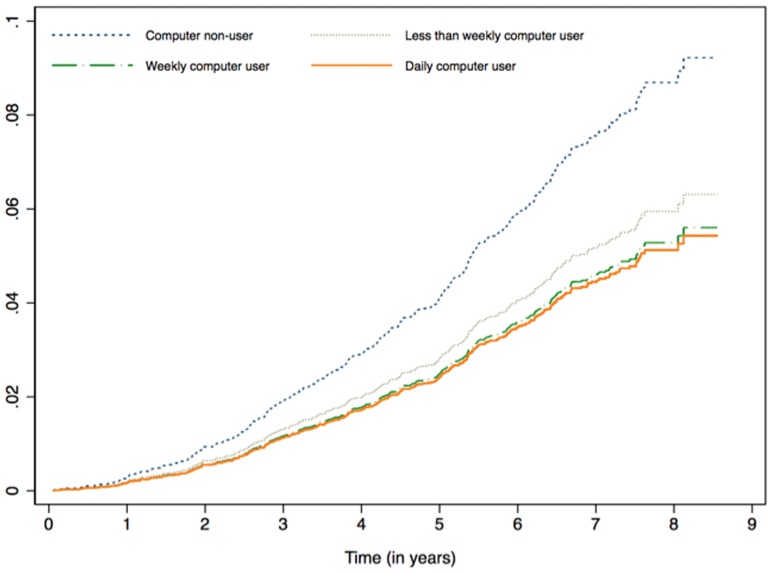
Cumulative hazard of dementia among older men according to their use of computers. The analysis was adjusted for age, high school attainment, social network, and presence of depression and significant clinical morbidity.

On average, we followed participants 6 years (SD = 1.7; range 6 months to 8.5 years), during which time 347 (6.3%) men received a diagnosis of dementia in WADLS. The crude IR of dementia for computer non-users and users was 13.3 (95%CI = 11.9–15.0) and 6.6 (95%CI = 5.2–8.2) per 1000 persons per year, respectively. HR of dementia amongst computer users was 0.49 (95%CI = 0.38–0.64) compared with non-users, and the protective effect of computer use remained significant after the analyses were adjusted for age, high school attainment, social network, and presence of depression and significant clinical morbidity (HR = 0.62, 95%CI = 0.47–0.81). We also investigated whether there was a dosage-response association between the hazard of dementia and computer use: we found that compared with computer non-users the adjusted hazard ratios of dementia associated with less than every week, at least weekly, and every day use were respectively 0.68 (95%CI = 0.41–1.13), 0.61 (95%CI = 0.39–0.94) and 0.59 (95%CI = 0.40–0.87) (Figure 1).

Table 2 shows the crude and adjusted HR of dementia according to the type of computer use activity. All activities assessed were associated with decreased hazard of dementia compared with no computer use.

**Table 2:** Incidence ratio (IR) and Hazard ratio (HR) of dementia according to type of computer activity.

Type of computer use		Dementia N = 347	IR (95%CI) per 1000 / year	Crude HR (95%CI)	Adjusted HR* (95%CI)
Email	Computer non-user, n (%)	289 (7.9)	13.3 (11.9–15.0)	1	1
	Computer user – no email, n (%)	39 (5.3)	8.8 (5.6–13.9)	0.7 (0.5–0.9)	0.7 (0.5–1.0)
	Email, n (%)	33 (3.0)	4.9 (3.1–8.0)	0.4 (0.3–0.5)	0.5 (0.4–0.8)
Games	Computer non-user, n (%)	289 (7.9)	13.3 (11.9–15.0)	1	1
	Computer user – no games, n (%)	39 (4.3)	7.1 (4.5–11.1)	0.5 (0.4–0.7)	0.6 (0.4–0.9)
	Games, n (%)	33 (3.5)	5.9 (3.7–9.5)	0.4 (0.3–0.6)	0.6 (0.4–0.9)
Internet	Computer non-user, n (%)	289 (7.9)	13.3 (11.9–15.0)	1	1
	Computer user – no internet, n (%)	43 (5.8)	9.8 (6.3–15.1)	0.7 (0.5–1.0)	0.8 (0.6–1.1)
	Internet, n (%)	29 (2.6)	4.3 (2.6–7.1)	0.3 (0.2–0.5)	0.5 (0.3–0.7)
Word processing	Computer non-user, n (%)	289 (7.9)	13.3 (11.9–15.0)	1	1
	Computer user – no word, n (%)	30 (5.0)	8.4 (5.1–13.7)	0.6 (0.4–0.9)	0.7 (0.5–1.0)
	Word processing, n (%)	42 (3.3)	5.6 (3.6–8.7)	0.4 (0.3–0.6)	0.6 (0.4–0.8)
Other	Computer non-user, n (%)	289 (7.9)	13.3 (11.9–15.0)	1	1
	Computer user – no other use, n (%)	43 (5.0)	8.3 (5.4–12.9)	0.6 (0.4–0.9)	0.7 (0.5–1.0)
	Other uses, n (%)	29 (2.9)	4.9 (3.0–8.1)	0.4 (0.3–0.5)	0.5 (0.3–0.8)

95%CI: 95% confidence interval of the ratio.

* Adjusted for age, high school attainment, social network, and presence of depression and significant clinical morbidity.

We then completed a sensitivity analysis in which we excluded 430 men who developed dementia or died during the initial 3 years of follow up (this analysis aimed to address the potential risk of reverse causality). The crude and adjusted HRs of dementia associated with computer use were 0.57 (95%CI = 0.43–0.77) and 0.72 (95%CI = 0.53–0.99).

### Calculating the HR of dementia after multiple imputation and adjustment for MMSE scores

We used multiple imputation techniques (n = 10) to estimate the MMSE scores of the 1618 men who provided information on computer use but did not complete the MMSE, and then re-ran our analyses to estimate the HR of dementia in the sample taking into account the cognitive performance of men at the baseline assessment (i.e., HIMS wave 2), in addition to age, high school attainment, social network, and presence of depression and significant clinical morbidity. The adjusted HR was 0.66 (95%CI = 0.50–0.86).

## Discussion

The results of this study indicate that the risk of incident dementia is about 30% to 40% lower among older computer users than non-users, and show that these findings cannot be attributed to age, education, social isolation, depression, poor physical health, or prevalent cognitive impairment. But before discussing how best to interpret these findings, we will consider the limitations of our study design.

### Limitations

The 5506 participants of this study consisted of a subsample of men whose health and activity levels were probably higher than those of the general population of similar age [23], [24], [25], although such a bias would most likely have moved the results towards the null hypothesis (as those who were sicker and had more cognitive impairment would be less likely to use computers). We also acknowledge that the implications of our findings for women remain uncertain as the proportion older Australian women who use computers is lower than that of men [9]. In addition, the observational nature of the study limits our ability to establish a causal relationship between computer use and lower risk of dementia or cognitive impairment, although our analyses indicate that such a link cannot be easily attributed to bias due to the inclusion of prevalent cases of dementia in the sample.

The validity of the diagnosis of dementia according to the WADLS has not been established, and although data linkage increases case-ascertainment compared with death certificates [26], the low prevalence of dementia in our sample suggest that its sensitivity is low (for example, cases of dementia diagnosed in the private sector are not recorded in WADLS). Hence, some men with dementia might have not been included in the sample of cases. We attempted to minimise the possibility of such a bias by conducting a sensitivity analysis and by using multiple imputation. The consistency of the effect-estimates derived from different approaches to the analysis of the data suggests that our results were not affected substantially by ascertainment bias.

Our data on computer use was self-reported, although the dosage response that we observed suggests that the results are most likely valid. Furthermore, confounding by unmeasured factors may have affected the results of our study. For example, computer use may be a surrogate marker for other behaviours, such as reading or ability to adopt new technologies, and despite our efforts to take relevant factors into account in our analyses, residual confounding may conceivably explain some of the association between computer use and risk of dementia (e.g., confounding due to physical or cognitive activity). In addition, we did not collect information about how long participants had been using computer for, and are therefore unable to comment whether more prolonged exposure to computer activities is an important factor in the modulation of dementia risk. Finally, we are unable to comment on whether computer use alters the progression of the pathological processes that lead to dementia or merely delay their clinical expression by improving the testing ability of older men.

### Interpretation

Tun and Lachman examined the cross-sectional association between computer use and cognitive performance among 2671 American adults aged 32 to 84 years (635 aged 65+ years) [10]. They found that the frequency of computer activity was directly associated with the cognitive scores of participants, particularly on tests assessing executive control [10]. Consistent with those findings, Small and colleagues showed, in a small cross-sectional functional MRI study, that internet browsing activates brain regions important for decision-making and complex reasoning, particularly in older adults who are regular internet users [27]. They also found that older participants who were not familiar with internet search routines showed no evidence of brain activation in brain regions not involved in reading [27], suggesting that repeated exposure to internet search may engage greater and more diverse regions of the brain.

Whilst it seems doubtful that cognitive training with computers enhances cognitive function over a short to medium period of time [28], [29], [30], there is virtually no information available on the likely effect of computer use over many years. Our findings show that, compared with computer non-use, computer use reduces the risk of dementia over a mean follow up period of 6 years. These results are consistent with currently available literature suggesting that participation in cognitive stimulating activities reduces the medium to long-term risk of dementia [6].

Animal models have uncovered numerous physiological pathways by which cognitive stimulation might decrease later cognitive decline [31], and preliminary epidemiological data suggest that environmental changes may already be leading to a reduction in the prevalence of dementia in the community. Lobo and colleagues observed a 25% reduction in the prevalence ratio of dementia in Zaragoza, Spain, between 1988–89 (n = 1080) and 1994–96 (n = 3715) [32]. This effect was most noticeable amongst men 70 to 85 years [32]. They suggested that such a drop in prevalence was most likely due to environmental causes. Our findings identify the use of computers as a novel environmental exposure that might potentially explain these findings. Should the latter explanation prove correct, then the increase in the number of cases of dementia over the next 40 years may not be as dramatic as is currently expected [33].

Given the limitations of our study design, our findings should be considered tentative. Sufficiently powered randomized trials that follow up older adults for several years (rather than months) would be required to establish with certainty the link between computer use and long term risk of dementia. However, given the ever-increasing use of computers by the community, the design of such a trial may be challenging, particularly in developed countries. In the meantime, there seems to be no obvious reason not to encourage older people to embrace the use of computer technology, as long as one remains mindful of the negative musculoskeletal and cardiovascular consequences of prolonged physical inactivity [34] and the many advantages of a balanced and healthy lifestyle [35].
